# Harmful Roles of TLR3 and TLR9 in Cardiac Dysfunction Developing during Polymicrobial Sepsis

**DOI:** 10.1155/2018/4302726

**Published:** 2018-09-30

**Authors:** Fatemeh Fattahi, Mark W. Russell, Elizabeth A. Malan, Michella Parlett, Elizabeth Abe, Firas S. Zetoune, Peter A. Ward

**Affiliations:** Department of Pathology, University of Michigan Medical School, Ann Arbor, MI 48109, USA

## Abstract

We determined the roles of TLR3 and TLR9 in adverse events of polymicrobial sepsis, with a focus on development of septic cardiomyopathy, progression of which we have recently shown to be complement- and histones-dependent. So Wt, TLR3-knocked out (K.O.), and TLR9-K.O. mice were subjected to polymicrobial sepsis following cecal ligation and puncture (CLP). In the absence of either TLR3 or TLR9, the intensity of echocardiogram (Echo)-Doppler dysfunction during development of cardiomyopathy was substantially reduced in the K.O. mice. Based on our prior studies emphasizing the adverse effects of plasma C5a and histones in the cardiomyopathy of sepsis, in TLR3- and TLR9-K.O. mice, there were striking reductions in plasma levels of C5a and histones as well as reduced levels of cytokines in plasma and heart tissue after CLP. Since we know that histones cause cardiac dysfunction, rat cardiomyocytes (CMs) were exposed* in vitro* to the histones (purified from calf thymus), which caused bleb formation on the surfaces of CMs, suggesting histones may perturb the cell membrane of CMs.* In vitro*, exposure of CMs to the histones for 3 hours caused lactate dehydrogenase release from CMs. These data indicate that sepsis-induced cardiac dysfunction requires presence of TLR3 and TLR9 and may be linked to histone-induced damage of CMs.

## 1. Introduction

 The history of toll-like receptors (TLRs) began with the seminal discovery in 1981 that antimicrobial peptides represent a key mechanism of innate host defense in insects [1, 2]. TLRs, a family of pattern recognition receptors, are a critical component of the innate immune system and responsible for the host defense against foreign pathogens via pathogen‐associated molecular pattern (PAMP) recognition [3, 4]. Exposure of immune cells to the ligands of these receptors activates intracellular signaling cascades that rapidly induce the expression of a variety of overlapping and genes involved in the inflammatory and immune responses [3]. Recent studies indicate that certain TLRs can also function as the sensors for endogenously produced ligands with danger‐associated molecular pattern (DAMP). DAMP ligands are often produced upon tissue stresses such as ischemia, hypoxia, or trauma [5, 6]. The heart retains an innate immune system that is intended to delimit tissue injury, as well as orchestrate homoeostatic responses, within the heart related to host defenses and to healing responses, especially after hypoxia. Several studies suggest that this intrinsic stress response system is mediated, at least in part, by the TLRs [5, 6]. Most TLRs are expressed on cell surfaces and recognize mainly microbial membrane components such as lipids, lipoproteins and proteins; another TLR group such as TLR3 and TLR9 are expressed exclusively in intracellular vesicles such as the endoplasmic reticulum, endosomes, lysosomes and endolysosomes, where they recognize nucleic acids related to damaged cells or to microbe presence. TLR3 was originally identified as the receptor for double‐stranded RNA, polyinosinic-polycytidylic acid (polyI:C), which mimics viral infection and induces antiviral immune responses. TLR9 recognizes DNA derived from both DNA viruses and bacteria [7].

It is known that TLR3 and TLR9 are expressed both* in vivo* in the mouse heart as well as* in vitro* by cardiomyocytes (CMs) [8]. The role of TLR3 and TLR9 were shown in different conditions/diseases leading to cardiac dysfunction and which was associated with protection against cardiac dysfunction in the absence of either TLR3 [9, 10] or TLR9 [11, 12]. Injurious responses in mouse hearts infected with the coxsackievirus are also TLR9-dependent [13].

The harmful effects of TLR3 or TLR9 on cardiac dysfunction during sepsis have been reported [10, 12]. TLR3^−/−^ mice showed preserved cardiac function after sepsis [10]. Also TLR9^−/−^ mice showed attenuated septic cardiomyopathy and a significant reduction of cardiac inflammation after sepsis [12]. Besides the inflammatory cytokines, new biomarkers, “extracellular histones” were recently shown by our group and others to play a major role in sepsis-related multiorgan dysfunction and death [14–17]. Histones are highly proinflammatory and prothrombotic [18]. We showed that extracellular histones are mainly released from neutrophils during sepsis after formation of neutrophil extracellular traps (NETs) which is C5a-dependent [14, 19, 20]. We have also shown that systemic histones neutralization mAb greatly attenuated the adverse effects of sepsis protecting the heart, resulting in enhanced survival after sepsis induced by cecal ligation and puncture (CLP) [14].

The complement system is also a critical component of the innate immune system and responsible for the host defense against foreign pathogens (bacteria, viruses, and fungi) [21, 22]. For several years, we have had a focus on dysfunction of the heart in sepsis and shown that such outcomes are linked to C5a and C5a receptors [14, 23–27]. Complement system like TLRs system can be activated rapidly to provide crucial first-line host defense and act as mediators between innate and adaptive immunity [28]. Previous studies have demonstrated that TLR3 and TLR9 mediate a systemic inflammatory response and contribute to cardiac dysfunction in animal models of polymicrobial sepsis [10, 12], but the downstream mechanisms causing these injuries are unknown. TLRs and complement were commonly studied as separate components in the host defense [29]; the crosstalk between these two components during polymicrobial sepsis and their possible interplay in sepsis-induced cardiac dysfunction is poorly understood [30]. There is one study showing activation of TLR2, TLR3, and TLR4 remarkably enhanced complement factor B synthesis and release by CMs* in vitro *[30]. We decided to study the role of TLR3 and TLR9 in the heart function during polymicrobial sepsis and proposed the role for complement C5a and histones mediating these events. For this aim we performed heart functional studies in TLR3^−/−^ and TLR9^−/−^, by applying Echo-Doppler technology after sepsis, to compare their responses with the septic Wt mice. We then measured the levels of proinflammatory cytokines (IL-6 and TNF), histones, and C5a in plasma from TLR3^−/−^, TLR9^−/−^, and Wt after inducing polymicrobial sepsis by CLP. We have also investigated the proinflammatory cytokines pattern in the heart tissue in these mice after inducing CLP.

## 2. Material and Methods

### 2.1. Animals and Anesthesia

Male C57BL/6 (Wt and TLR3^−/−^) mice were purchased from the Jackson Laboratory (Bar Harbor, ME). Male BALB/c (Wt and TLR9^−/−^) mice were a donation from Theodore J. Standiford's lab, Division of Pulmonary and Critical Care Medicine, University of Michigan Medical Center. Mice (8 to 12 weeks old, 25–30g) were housed 5 per cage under conditions that were specific pathogen-free. Male pathogen-free Sprague-Dawley rats (250–300 g) were purchased from Harlan Laboratories (Indianapolis, IN). Animals were provided with a standard food diet and water. All animals were maintained according to protocols approved by University of Michigan Committee on the Use and Care of Animals and in accordance with the National Institutes of Health institutional guidelines. All animals were anesthetized using the combination of ketamine (Hospira, Lake Forest, IL) and xylazine (Lloyd Laboratories, Shenandoah, IA) intraperitoneally. Minimum five mice were used in each experiment.

### 2.2. Experimental Sepsis

Cecal ligation and puncture (CLP) procedure was used for inducing sepsis in mice as described previously [31]. For this study mid-grade CLP was used, which results in ~50% survival over 7 days. Sham animals underwent the same procedure, including manipulation of the bowel in the absence of CLP. All animals (control sham and CLP) received fluid resuscitation (1 ml saline, given subcutaneously in the nuchal region). The animals were euthanized at 8 hours (hr.) after CLP.

### 2.3. Blood and Heart Collection

At 8 hr. after the induction of CLP, anticoagulated blood (using acid citrate dextrose; ACD) was taken by cardiac puncture for collecting plasma. Then the hearts were excised, rinsed in ice-cold PBS, and was mechanically homogenized in RIPA lysis buffer (Millipore Sigma, Burlington, MA) containing protease inhibitor cocktail (Roche Diagnostics GmbH, Mannheim, Germany). Supernatants containing cytosolic protein were collected after centrifugation and used to detect the intracardiac cytokines. Myocardial cytokine levels were normalized to the protein concentration. Total protein estimations were determined by the bicinchoninic acid (BCA) assay (Sigma-Aldrich, St. Louis, MO) according to the manufacturer's protocol.

### 2.4. Isolation of CMs

The isolation of adult rat CMs was performed using a Langendorff perfusion system, as we previously described [14, 25, 26]. The hearts were retrograde perfused with enzyme solution, according to manufacturer's directions (Liberase; Hoffmann-La Roche, Mannheim, Germany). Following digestion, the heart was detached from the Langendorff apparatus, atria and vessels were removed, and the ventricles were cut into small pieces, which were gently triturated with a plastic transfer pipette. After isolation of the CMs, the Ca^2+^concentration in the buffer fluid was gradually increased (to 1.8 mM), and the cells were cultured in M199 medium with 1% insulin-transferrin-selenium-X (Gibco; Life Technologies, Carlsbad, CA) and antibiotic-antimycotic (Invitrogen, Carlsbad, CA).

### 2.5. Enzyme-Linked Immunosorbent Assays (ELISAs)

The levels of the TNF and IL-6 cytokines and complement C5a were measured in plasma taken from the Wt, TLR3^−/−^, and TLR9^−/−^ mice by ELISA using DuoSet sandwich ELISA kits (R&D Systems, Minneapolis, MN). Cytokines levels were also measured in supernatants from heart homogenates from Wt, TLR3^−/−^, and TLR9^−/−^ mice using DuoSet sandwich ELISA kits, according to the manufacturer's protocol. The levels of cytokines in the heart tissue were expressed as pg/mg heart tissue.

### 2.6. Histone ELISA

Histones levels in mouse plasma were measured by using a cell death detection ELISA kit (Sigma-Aldrich, St. Louis, MO) which detects all individual histones. A histone mixture (containing all individual histones of H1, H2A, H2B, H3, and H4) was used to establish a standard curve, as we described previously [14, 32].

### 2.7. Lactate Dehydrogenase (LDH) Cytotoxicity Assay

LDH assay was used to detect the cytotoxicity levels of histones on rat CMs according to the protocol which we described before [33]. Briefly, supernatants fluids were obtained from CMs exposed to histone, using phenol red-free media (Gibco, Grand Island, NY). The percentage of cytotoxicity (LDH release) from the sample treated with histone was measured compared to LDH content in total lysis fluids induced by 0.1% triton detergent (Sigma-Aldrich, St. Louis, MO). LDH release was measured using LDH assay kit (Cayman Chemical, Ann Arbor, MI) according to the manufacturer's instructions.

### 2.8. Reagents

The following reagent/kit was used: purified histones from calf thymus and chemicals used for preparation of solutions for CM isolation were purchased from Sigma-Aldrich (St. Louis, MO). Endotoxin contamination of the histone preparations was <0.02 EU/mg using LAL assay (Lonza, Allendale, NJ) [33].

### 2.9. Confocal Imaging

For confocal imaging, CMs were cultured on sterile glass coverslips precoated with Poly-L-Y-Lysine (Sigma-Aldrich, St. Louis, MO). Cells were incubated with CellMask Deep Red Plasma membrane Stain (Invitrogen, Carlsbad, CA) after 30-minute treatment with the agonist. Confocal imaging was performed with a Zeiss LSM 510 Confocal microscope (Zeiss USA, Pleasanton, CA).

### 2.10. Transthoracic Echocardiography in Mice

Echocardiograms (Echos) were performed as previously described [14, 25, 26]. Briefly, all Echos were performed by a registered echocardiographer who was blinded to experiment details. Mice were weighed and anesthetized with inhaled isoflurane. Imaging was performed according to the recommendations of the American Society of Echocardiography using a Vevo 770 Micro-imaging system (Visualsonics Inc.) equipped with an RMV707B (15-45 MHz) transducer. Left ventricular (LV) area and LV length was measured from the parasternal long axis view and used to calculate the LV end systolic and diastolic volumes as follows: V = 4/3 x LV Area x LV length. The volumes at end systole (VolS) and end diastole (VolD) were used to calculate stroke volume (SV = VolD – VolS) and ejection fraction (EF% = SV / VolD x 100). Cardiac output (CO = SV x heart rate) was calculated from stroke volume and heart rate. Mitral valve E and A wave inflow velocities were sampled at the tips of the leaflets of the mitral valve from the apical four chamber view. Doppler tissue imaging was performed with acquisition of peak E' velocity from the lateral (E'la) and septal annulus (E'sa) of the mitral valve imaged from the apical four chamber view. Isovolumic relaxation time, from the closure of the aortic valve to the opening of the mitral valve, was measured from the apical five chamber view using Doppler flow imaging. Imaging was performed at 8 hr. after CLP.

### 2.11. Statistical Analysis

All values were expressed as means ± SEM. Data were analyzed and graphed using GraphPad Prism software (GraphPad version 7, La Jolla, CA). Significant differences between two independent groups were determined, using independent student's* T* test and between more than 2 groups using one-way ANOVA followed by Dunnett's or Tukey's multiple comparison test. Differences were considered significant when p< 0.05.

## 3. Results

### 3.1. Echo-Doppler Parameters in Septic Mice (Wt, TLR3^−/−^, TLR9^−/−^)(Figures 1 and 2)

**Figure 1:**
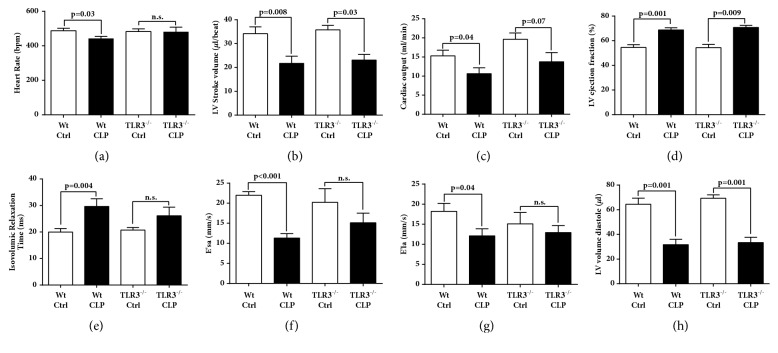
Echo-Doppler parameters in Wt and TLR3^−/−^ mice 8 hr. after CLP. Heart rate (a), left ventricular (LV) stroke volume (b), cardiac output (c), LV ejection fraction (d), isovolumic relaxation time (e), peak E' velocity from the septal annulus, E′sa (f), peak E' velocity from the lateral annulus, E'la (g), and LV volume diastole (h) represent selected measures of systolic and diastolic heart function in mice before and 8 hr. after CLP. For each bar, n = 5 mice. n.s., nonsignificant.

**Figure 2:**
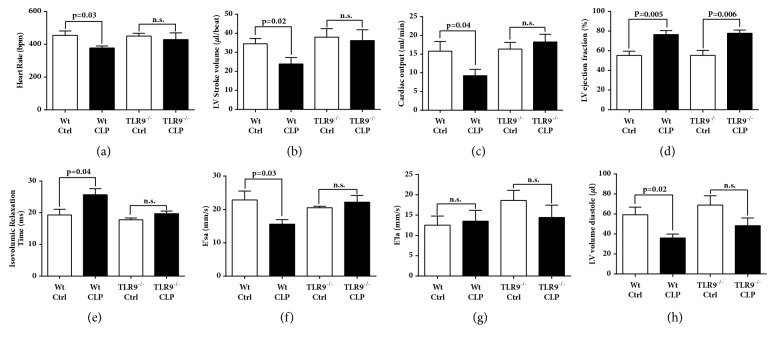
Echo-Doppler parameters in Wt and TLR9^−/−^ mice 8 hr. after CLP. Heart rate (a), left ventricular (LV) stroke volume (b), cardiac output (c), LV ejection fraction (d), isovolumic relaxation time (e), peak E' velocity from the septal annulus, E′sa (f), peak E' velocity from the lateral annulus, E'la (g), and LV volume diastole (h) represent selected measures of systolic and diastolic heart function in mice before and 8 hr. after CLP. For each bar, n = 5 mice. n.s., nonsignificant.

To examine the contribution of the toll-like receptors, TLR3 and TLR9, to the* in vivo* functional changes in cardiovascular performance developing after CLP, Echo-Doppler was obtained 8 hr. after CLP in TLR3^−/−^ (Figure 1), in TLR9^−/−^ (Figure 2) mice and in control Wt littermate mice. As has been noted previously [14, 25, 26], mice subjected to CLP demonstrated significant abnormalities of systolic (frames (a)-(d) in Figures 1 and 2) and diastolic (frames (e)-(h) in both figures). There were important hemodynamic differences applied by Echo-Doppler technology related to TLR3^−/−^ and TLR9^−/−^ mice after CLP, suggesting that both receptors have important but different contributions to cardiac performance in response to sepsis. In response to CLP, both TLR3^−/−^ and control Wt mice demonstrated significant decreases in stroke volume and cardiac output and a significant increase in ejection fraction (frames (a)-(h)). A modest reduction in heart rate developing in control Wt was not seen in TLR3^−/−^ septic mice, but all other systolic measures were very similar. However, there were some subtle differences in measures of diastolic performance between in the TLR3^−/−^ and control Wt. While control Wt animals demonstrated a significant increase in isovolemic relaxation time and significant increases in tissue relation velocities (E'la and E'sa) and LV end diastolic volume, TLR3^−/−^ mice demonstrated similar trends in diastolic performance after CLP but only the increase in LV end diastolic volume reached statistical significance.

Unlike the TLR3^−/−^ mice, TLR9^−/−^ (Figure 2) mice demonstrated only modest hemodynamic changes in responses to CLP. Most measures of systolic and diastolic performance in TLR9^−/−^ mice were equivalent to those in nonseptic mice. The only notable hemodynamic changes in the TLR9^−/−^ mice after CLP was an increase in the LV ejection fraction and trend towards reduced LV end diastolic volumes. These suggest an important role for TLR9 in mediating the hemodynamic effects of CLP. While TLR3 receptor may participate in responses, our studies would suggest that their contribution is more modest while absence of TLR9 prevents most of the Echo-Doppler abnormalities developing after CLP when compared to responses of Wt mice.

### 3.2. Plasma Levels of C5a and Histones in Wt, TLR3^−/−^, and TLR9^−/−^ 8 hr. after CLP (Figure 3)

**Figure 3:**
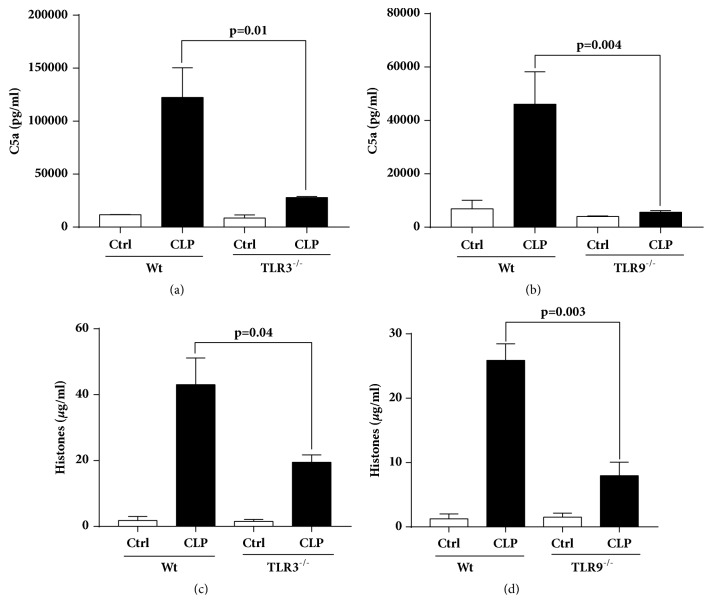
Plasma levels of C5a and extracellular histones in TLR3^−/−^ and TLR9^−/−^ mice compared to their Wt mice 8 hr. after CLP.

In Figure 3, we determined plasma levels of C5a and histones (by ELISA) 8 hr. after CLP in Wt, TLR3^−/−^, and TLR9^−/−^ mice, using the 8 hr. time period point which we know correlates with the cardiomyopathy of sepsis based on Echo-Doppler endpoints [14, 25, 26]. For each bar in Figure 3, number of the mice is ≥ 5 mice. As shown in frame (a), these were dramatic reductions in levels of plasma C5a when compared to levels in Wt mice (77% reduction in TLR3^−/−^ mice [frame (a)] and 87% reduction in TLR9^−/−^ mice [frame (b)]). It appears that C5a responses of septic mice were dramatically reduced in mice lacking TLR3 or TLR9. Reasons for these reductions are not known, since there is no definitive evidence on how sepsis causes complement activation resulting in C5a generation.

In frames (c) and (d), plasma histones were measured (by ELISA) in plasma 8 hr. after CLP, in Wt, TLR3^−/−^, and TLR9^−/−^ mice. We have recently shown that plasma histones play an important role in the adverse events of sepsis including cardiomyopathy [14]. In these studies, plasma histones in TLR3^−/−^ mice were reduced by 54% when compared to Wt mice and by 69% in TLR9^−/−^ mice. Our recent data suggest that sepsis-induced generation of C5a which reacts with neutrophils containing C5a receptors, results in PMN activation, formation of NETs, and release of extracellular histones [14, 19, 20].

### 3.3. Effects of TLR3 or TLR9 Absence on Plasma Cytokines in Septic Mice (Figure 4)

**Figure 4:**
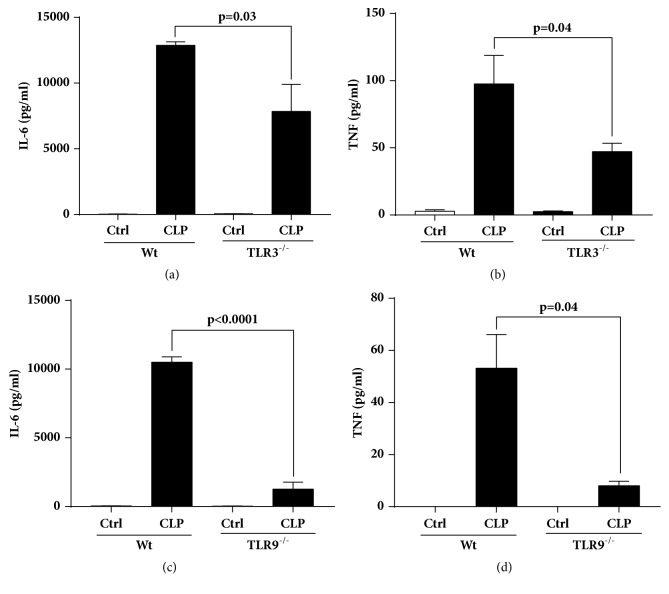
Levels of proinflammatory cytokines in plasma from TLR3^−/−^ (a, b) and TLR9^−/−^ (c, d) mice compared to their Wt 8 hr. after inducing CLP.

We extended the studies to determine how absence of TLR3 (frames (a) and (b)) or TLR9 (frames (c) and (d)) in CLP mice affects sepsis-induced appearance of plasma proinflammatory cytokines. The data in Figure 4 show that plasma levels of IL-6 fell by 40% in TLR3^−/−^ septic mice (frame (a)) and 52% in TLR9^−/−^ septic mice (frame (c)) and absence of TLR3 or TLR9 resulted in reductions of plasma TNF by 50% (frame (b)) and 84% (frame (d)) respectively, compared to their Wt septic mice. While these data are preliminary, they indicate that TLR absence reduces plasma levels of proinflammatory cytokines, suggesting that events that “drive” the adverse consequences of sepsis are negatively affected by absence of TLR3 or TLR9.

### 3.4. Effects of TLR3 or TLR9 Absence on Heart Cytokines Content in Septic Mice (Figure 5)

**Figure 5:**
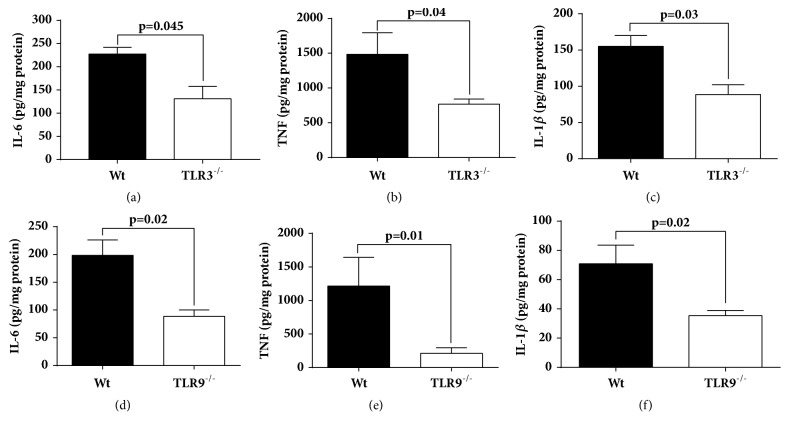
Levels of proinflammatory cytokines in the heart tissue from TLR3^−/−^ (a-c) and TLR9^−/−^ (d-f) mice compared to their Wt 8 hr. after inducing CLP.

We measured the levels of proinflammatory cytokines in cardiac tissue using heart homogenate to study how the absence of TLR3 or TLR9 affects the appearance of proinflammatory cytokines in the heart tissue after sepsis. As shown in Figure 5, the levels of the proinflammatory cytokines in TLR3^−/−^ mice are significantly lower compared with the Wt at 8 hr. after inducing sepsis by more than 40% (42% in IL-6, 48% in TNF, and 43% in IL-1*β* levels) (frames (a)-(c)). In the case of TLR9^−/−^, the levels of proinflammatory cytokines in the heart tissue were remarkably lower compared to the Wt mice (55% in IL-6, 80% in TNF, and 50% in IL-1*β* levels) (frames (d)-(f)).

### 3.5. Evidence that Histones Cause Defects in CMs Exposed In Vitro to the Histones (Figure 6)

**Figure 6:**
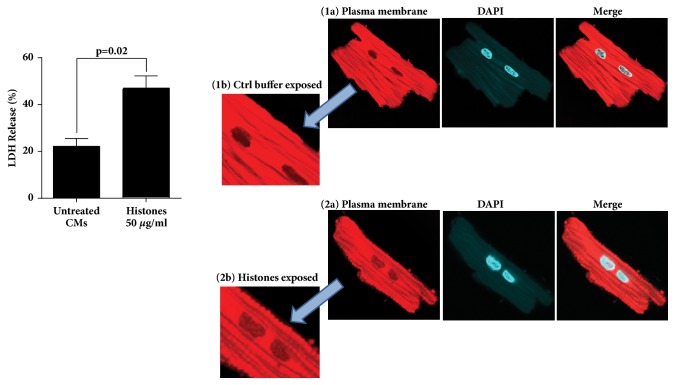
Lactate dehydrogenase (LDH) release from rat cardiomyocytes after* in vitro* exposure to purified histones (50 *μ*g/ml) for 3 hr. at 37°C (left panel). Morphological evidence of damage to rat cardiomyocytes (bleb formation) after the exposure to the purified histones (50 *μ*g/ml) for 30 minutes at 37°C (right panel). Bleb formation was detected by Deep Red Plasma Membrane Stain.

Since we know that circulating histones in septic mice are associated with CM dysfunction [14], two* in vitro* studies were completed. In Figure 6, CMs were isolated from normal rat hearts using established techniques described previously [14, 25–27] and then incubated for 3 hr. at 37°C with buffer (vehicle control) or with the histones (50 *μ*g/ml). This concentration approaches levels of plasma histones levels in septic Wt mice [14]. As is apparent in Figure 6, CMs incubated in buffer for 3 hr. at 37°C released some LDH, which approached 20% of total LDH in CMs. However, in the presence of the histones, almost 50% of the total LDH in CMs was released, indicating that the histones cause damage to CMs. The reason for LDH release from buffer-exposed CMs is probably due to the isolation and culture process. The process of isolating adult CMs carries an inherent risk of causing cellular damage, activation of stress response pathways [34], which can lead to reducing cell viability. In fact, the CMs isolation technique requires a long procedure and needs digestive enzymes to free up the CMs and reintroducing them into a calcium-containing medium after isolation. For LDH assay experiment, we had to incubate these CMs with buffer or histones for 3 hr. which all may result in releasing LDH.

We have also found the morphological evidence of damage to CMs after exposure to the histones. Based on the LDH data in in the left panel in which exposure of CMs to the histones caused LDH release, in the right panel, we studied the morphological changes in the rat CMs after incubating with the histones (50 *μ*g/ml), for 30 minutes at 37°C. We then exposed the CMs to CellMask Deep Red Plasma membrane stain for 10 minutes at 37°C and washed and examined by light microscopy for morphological changes, with a focus on the cell membrane. The CellMask™ Plasma Membrane Stains allow fast and uniform labeling of the plasma membrane without the cell-type differences exhibited by lectins and is a lipophilic fluorophore which is an excellent live and fixed cell membrane stain for cellular plasma membranes [35–38]. The red stain has been used to detect changes in the basement membrane of various cell types, especially the presence of blebs in the cell membrane. The CMs exposed to buffer (vehicle control) showed smooth cell membranes with no evidence of bleb formation (frames 1a, 1b in the right panel). In striking contrast, CMs exposed to the histones showed diffuse numerous blebs on the cell membranes (frames 2a, 2b in the right panel). There was some evidence in CMs exposed to the histones that blebs associated with the cell membrane were being released. We believe these data show the ability of the histones to induce significant bleb formation in CM cell membranes, with occasional release of blebs, indicating adverse changes in the CM cell membranes as a result of exposure to the histones. Such events appear to correlate with release of LDH shown in the left panel. It is not known if C5a induce similar changes in CM cell membranes.

## 4. Discussion

In the setting of polymicrobial sepsis, most studies in the past investigated the role of TLR2 and TLR4 as mediating a systemic inflammatory response. In general these studies have shown that TLR2 and TLR4 contribute to high mortality and multiorgan dysfunction in animal models of polymicrobial sepsis [39–42]. There is also limited evidence suggesting protective effects and decreased mortality in mice that lack either of the TLR3 or TLR9 receptor after CLP [10, 43, 44]. Our functional data measuring Echo-Doppler parameters (Figures 1 and 2) show in the absence of either TLR3 or TLR9 the intensity of cardiac dysfunction during development of septic cardiomyopathy was substantially reduced in K.O. mice, especially in TLR9^−/−^ mice. These data suggest that TLR9 and, with more modest effect, TLR3 involvement play important roles in mediating the hemodynamic effects of CLP. Our data are in line with the published reports in which TLR3^−/−^ mice showed significant attenuation of cardiac dysfunction or myocardial necrosis after myocardial infarction or during ischemia-reperfusion injury [45, 46]. TLR3^−/−^ mice are shown to have more survival after inducing polymicrobial sepsis and showed a maintenance of cardiac function at pre-CLP levels [10]. Decreased CLP-induced cardiac myocyte apoptosis and attenuated CLP-induced Fas and FasL expression in the myocardium of TLR3^−/−^ mice were proposed as the underlying mechanism by the investigators [10]. TLR9 also appeared to play a role in myocardial inflammation as well as defective cardiac contractility [47]. TLR9^−/−^ mice showed increased survival after CLP compared to the Wt which was associated with enhanced clearance of bacteria from the blood and peritoneal cavity, as well as a dramatic decrease in serum inflammatory cytokines [44]. In line with the mentioned findings from TLR3^−/−^ and TLR9^−/−^ mice, our data in Figures 3–5 show remarkably less intensified inflammatory pattern, extracellular histone released, and C5a production in these K.O. mice compared to the Wt mice during sepsis.

It has been reported that signaling pathways upon stimulation of TLRs (including TLR3 and TLR9) in variety of cell types (e.g., lymphocytes, macrophages, and dendritic cells) result in activation of a series of signaling proteins in which expression of proinflammatory cytokine and chemokine genes is increased [48]. Our data showing lower levels of proinflammatory cytokines in the K.O. mice suggest that signaling through TLR3 and TLR9 involves release of these proinflammatory cytokines during sepsis. Moreover, the lower levels of complement C5a and extracellular histones in plasma during sepsis in TLR3^−/−^ and TLR9^−/−^ mice suggest that these biological markers may act as a downstream effector of TLR3 and TLR9 signaling. This is an important finding since complement activation product, C5a, is a part of the innate immune system components although C5a protects the host against infectious organisms as well as noninfectious agents via innate immune pathways [29], but excessive production of C5a during sepsis results in a series of harmful consequences [49]. Extracellular histones may also cause a series of responses that are tissue damaging or prothrombotic during sepsis [14, 15, 18], as also shown here damage in CMs after exposure to histones (Figure 6). In the past we have shown protective effects of the histone antibody (clone BWA3) against hearts dysfunction during polymicrobial sepsis. Cardiac function parameters in septic mice were improved with using this antibody, as measured by Echo-Doppler [14]. Although it is still unknown about the interplay between extracellular histones and TLR3 but it has been shown that extracellular histones function as DAMPs through TLR9 after ischemic injury to initiate inflammation [50, 51]. Our data indicate that both TLR3 and TLR9 contribute to systemic inflammatory responses and multiorgan dysfunction in which the production of extracellular histones and C5a are increased mediating septic cardiomyopathy in mice following CLP.

## Data Availability

All data generated or analysed during this study are included within the article. Our prior studies to support some of our data (Echo-Doppler data) are cited at relevant places within the text as references [14, 25–27].
